# Improving Outcomes of Tyrosine Kinase Inhibitors in Hepatocellular Carcinoma: New Data and Ongoing Trials

**DOI:** 10.3389/fonc.2021.752725

**Published:** 2021-10-11

**Authors:** Lisha Mou, Xiaohe Tian, Bo Zhou, Yongqiang Zhan, Jiao Chen, Ying Lu, Jing Deng, Ying Deng, Zijing Wu, Qi Li, Yi’an Song, Hongyuan Zhang, Jinjun Chen, Kuifeng Tian, Yong Ni, Zuhui Pu

**Affiliations:** ^1^ Department of Hepatopancreatobiliary Surgery, Shenzhen Institute of Translational Medicine, The First Affiliated Hospital of Shenzhen University, Shenzhen Second People’s Hospital, Shenzhen, China; ^2^ Shenzhen Xenotransplantation Medical Engineering Research and Development Center, Shenzhen Institute of Translational Medicine, The First Affiliated Hospital of Shenzhen University, Shenzhen Second People’s Hospital, Shenzhen, China; ^3^ Rausser College of Natural Resources, University of California, Berkeley, Berkeley, CA, United States; ^4^ College of Engineering, Boston University, Boston, MA, United States; ^5^ Faculty of Science, University of Waterloo, Waterloo, ON, Canada; ^6^ Imaging Department, Shenzhen Institute of Translational Medicine, The First Affiliated Hospital of Shenzhen University, Shenzhen Second People’s Hospital, Shenzhen, China; ^7^ The Faculty of Arts and Sciences, The University of British Columbia, Kelowna, BC, Canada

**Keywords:** sorafenib, lenvatinib, tyrosine kinase inhibitors, TKIs, hepatocellular carcinoma, HCC, targeted therapy

## Abstract

Targeted therapies such as oral tyrosine kinase inhibitors (TKIs) are the main therapeutic strategy effective for advanced hepatocellular carcinoma (HCC). Currently six tyrosine kinase inhibitors for HCC therapy have been approved. The newly approved first-line drug donafenib represent the major milestones in HCC therapeutics in recent years. However, drug resistance in HCC remains challenging due to random mutations in target receptors as well as downstream pathways. TKIs-based combinatorial therapies with immune checkpoint inhibitors such as PD-1/PD-L1 antibodies afford a promising strategy to further clinical application. Recent developments of nanoparticle-based TKI delivery techniques improve drug absorption and bioavailability, enhance efficient targeting delivery, prolonged circulation time, and reduce harmful side effects on normal tissues, which may improve the therapeutic efficacy of the TKIs. In this review, we summarize the milestones and recent progress in clinical trials of TKIs for HCC therapy. We also provide an overview of the novel nanoparticle-based TKI delivery techniques that enable efficient therapy.

## 1 Introduction

**Figure 1:**
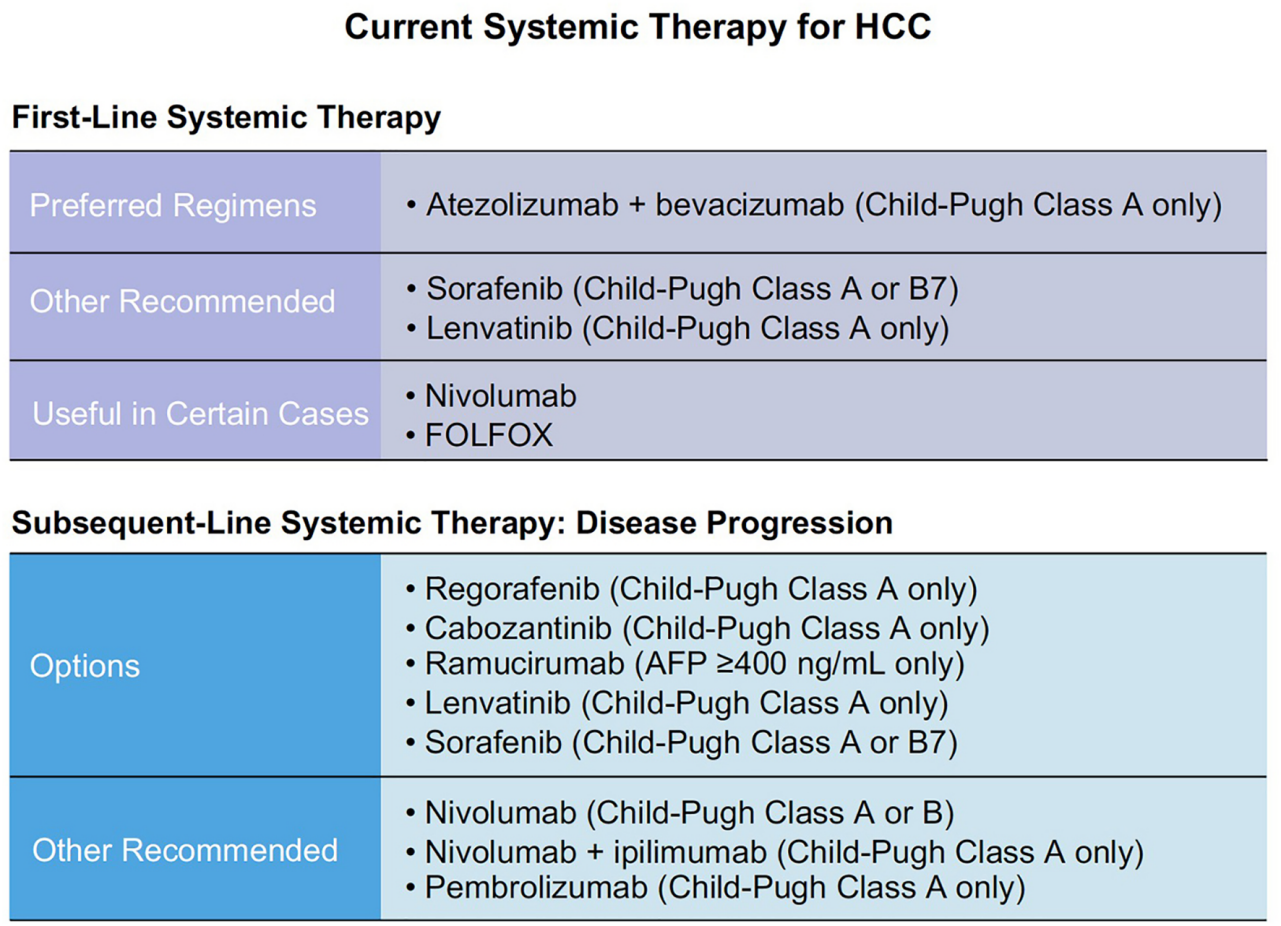
Current systemic therapies for HCC.

According to GLOBOCAN 2020 statistics, estimated liver cancer summed up to 905,677 new cases and 830,180 deaths in 2020 worldwide (1). Hepatocellular carcinoma (HCC) is the most common type of primary liver cancer. It occurs in approximately 85% of cirrhosis cases (2). The first tyrosine kinase inhibitor (TKI) was approved early this century as a potential precision therapy for HCC. Its specificity to targets made it more efficient and safer compared to traditional chemotherapies due to minimal impact on normal cells (3). Current systemic therapies for HCC are mainly based on TKIs, anti-angiogenesis drugs, and immunotherapy agents (**Figure 1**).

Since sorafenib was first approved for HCC and established its pioneer role in the field, a total of six TKIs have been approved for the treatments of HCC by regulatory authorities around the world. First-line drugs include sorafenib, lenvatinib, and the newly approved donafenib by Chinese National Medical Products Administration (NMPA). Second-line drugs are regorafenib, cabozantinib, and NMPA approved apatinib (**Table 1**) (4).

**Table 1:** Summary of approved TKIs.

No	Drug	Brand	Targets	Approved time/Organization
1	Sorafenib	Nexavar/Bayer	VEGFR1-3, TIE2, PDGFR, FGFR, BRAF, CRAF, KIT, FLT-3	2007/FDA
2	Regorafenib	Stivarga/Bayer	VEGFR1-3, TIE2, PDGFR, FGFR, BRAF, KIT, RET	2017/FDA
3	Lenvatinib	Lenvima/Eisai Inc	VEGFR1-3, PDGFR, FGFR1-4, RET, KIT	2018/FDA
4	Cabozantinib	Cometriq,Cabometyx/Exelixis Inc	VEGFR1-3, MET, ROS1, RET, AXL, NTRK, KIT	2019/FDA
5	Apatinib	Aitan/Jiangsu Hengrui	VEGFR2, KIT, RET, c-Src	2020/NMPA
6	Donafenib	Zepsun/Suzhou Zelgan	PDGFR, VEGFR, Raf	2021/NMPA

**Figure 2:**
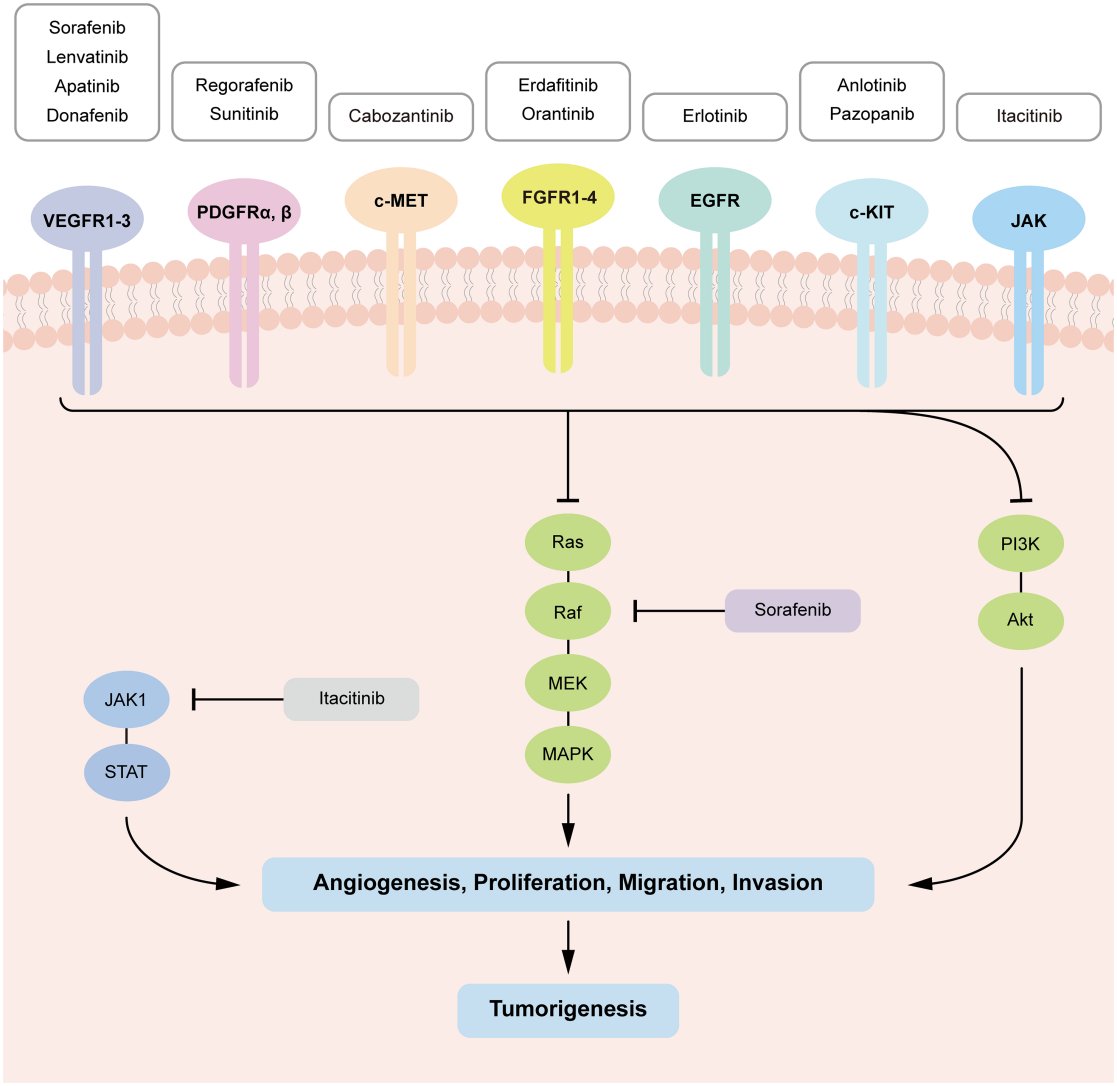
Tyrosine kinase-mediated signaling pathways in HCC tumorigenesis and targets of the TKIs. TKIs bind with corresponding kinases from phosphorylating tyrosine residues in their substrates, which inhibit downstream signaling pathways (RAS–MAPK, PI3K–AKT and JAK–STAT) activation, preventing cell proliferation, migration, invasion, and angiogenesis in HCC.

Other existing TKIs which were previously approved for the treatment of other cancer types are now under clinical trials for HCC, including sunitinib, erdafitinib, erlotinib, anlotinib, pazopanib an so on (**Table 2**). Most common targets of TKIs in HCC include vascular endothelial growth factor receptors (VEGFR), platelet-derived growth factors (PDGF), and tyrosine-protein kinases (5). Thus, TKIs would inhibit activation of corresponding signaling pathways through binding irreversibly or reversibly to various sites during the initial activation of receptor tyrosine kinases (RTKs) (6). This further prevents tumor growth and metastasis by preventing downstream signaling pathways from being activated (4, 7). TKIs which are discussed in the review are summarized in **Figure 2**.

**Table 2:** Representative clinical trials of tyrosine kinase inhibitors in hepatocellular carcinoma treatment.

Registered Trial Number	Duration(start-end)	Enrollment	Phase	Treatment	Targets	mPFS (months)	mOS (months)	ORR (%)	Adverse Events
NCT02645981 (ZGDH3)	2016.03 -2019.12	668	III	Donafenib *vs*. Sorafenib	VEGFR, PDGFR	3.7 *vs*. 3.6	12.1 *vs.* 10.3	4.6 *vs.* 2.7	hand-foot skin reaction, aspartate aminotransferase increased, blood bilirubin increased, platelet count decreased, and diarrhea
NCT01164202	2010.07 - 2017.07	78	II/III	Sunitinib + Doxorubincin-TACE	VEGFR, PDGFR, c-KIT, FLT3, RET	9.05	25	6	Hematologic toxicity, fatigue, transaminase elevation, hand-foot syndrome events
NCT03006926 (KEYNOTE 524)	2017.02 - 2019.10	104	Ib	Lenvatinib + Pembrolizumab	VEGFR, PDGFR	9.3	22	46	hypertension, diarrhea, fatigue, decreased appetite, hypothyroidism, hypertension, and Leukopenia/neutropenia
NCT03463876 (RESCUE)	2018.02 - 2020.02	190	II	Apatinib + Camrelizumab	VEGFR	5.7	N/A	34.3	hypertension, increased aspartate aminotransferase, proteinuria, hyperbilirubinemia, increased gammaglutamyltransferase, and neutropenia
ChiCTR-IPR-17012667	2018.01 - 2020.01	80	IV	Apatinib+TACE *vs* TACE	VEGFR	17.2 *vs* 12.5	N/A	55 *vs* 32.5 a	Fatigue, hand-foot syndrome, diarrhea, hypertension, proteinuria, oral ulcer
NCT04172571	2018.12 - 2020.06	30	Ib/II	Anlotinib + AK105	VEGFR, FGFR PDGFR, c-KiT, Ret	N/A	N/A	24	AST, increased ALT, asthenia, decreased platelet count, increased blood bilirubin, increased bilirubin conjugated, and rash
NCT02809534 (ALTER0802)	2016.09 - 2017.10	60	II	Anlotinib (with *vs.* without previous TKIs therapy)	VEGFR, FGFR PDGFR, c-KiT, Ret	80.8% *vs.* 72.5% b	20.1 *vs.* 7.9	N/A	hypertension, hypothyroidism, fatigue, hand-foot syndrome, elevated bilirubin, and diarrhea

a, ORR in three months; b,12-week mPFS rate; N/A, not available.

## 2 Different Kind of TKIs for HCCs

### 2.1 VEGFR-TKIs

#### 2.1.1 Sorafenib

Sorafenib was the first approved TKI for the treatment of advanced primary HCC by the FDA in 2007 (8). Sorafenib was first developed for the treatment of renal cell carcinoma and works through a dual mechanism, by targeting the serine/threonine kinase Raf or blocking autophosphorylation of multiple RTKs-VEGFR1, 2 and 3, PDGFR, c-Kit, and RET (9).

Sorafenib was termed the preferred first line therapy until recently replaced by the combination of atezolizumab and bevacizumab in 2021 according to the latest version of NCCN guidelines (10). In patients with unresectable hepatocellular carcinoma (HCC), the combination of atezolizumab and bevacizumab improved median progression-free survival (PFS) and overall survival (OS) (6.8 months *vs* 4.3 months) compared with sorafenib in the IMbrave150 trial (11).

Recently, studies on combination therapies based on sorafenib showed exciting results. The combination of sorafenib and transcatheter arterial chemoembolization (TACE) yielded an improved overall response rate (ORR) (55.9% *vs* 37.3%) and disease control rates (86.4% *vs* 67.8%) compared to TACE group (12). However, the sorafenib plus TACE therapy had increased AE. Besides, conflicting results were reported in other studies (ISRCTN93375053, NCT02425605) (13, 14). As a result, more studies are required to verify these findings.

#### 2.1.2 Lenvatinib

Lenvatinib, which was approved by FDA in 2018, is another significant first-line TKI that differs from sorafenib in its mechanisms (15). It is developed by Eisai Inc and inhibits angiogenesis targeting a broad range of receptors, including VEGFR1-3 and PDGFRα, β (16). In addition, it inhibits tumor cell proliferation *via* inhibition of the proto-oncogenes KIT and RET (17). The alternative targets that differentiate lenvatinib from other HCC TKIs are the fibroblast growth factor receptors (FGFR) 1-4, which also contributes to angiogenesis in the progression of tumor growth and metastasis (18). Lenvatinib binds VEGFR2 at its ATP mimetic quinoline moiety to the ATP binding site and the neighboring region *via* a cyclopropane ring. In comparison to the other licensed VEGFR2 kinase inhibitors, these findings indicate that lenvatinib has a different binding mode of interaction (19).

Despite its role in first-line drugs for advanced HCC, lenvatinib is also considered a better alternative to TACE when treating patients in the BCLC intermediate stage with a high tumor burden. These patients were thought to be susceptible to decline in liver function and poor therapeutic response. A recent study compared the median PFS in TACE-refractory patients that were treated with lenvatinib (5.8 months), sorafenib (3.2 months), and TACE (2.4 months). This indicates treatment with lenvatinib in replacement of TACE has the potential to acquire good therapeutic responses while preserving normal liver function (20).

In terms of combination therapies, lenvatinib plus pembrolizumab (an anti–PD-1 antibody) showed encouraging results with improved ORR and DCR for unresectable HCC (NCT03006926, **Table 2**) (21). The FDA granted lenvatinib plus pembrolizumab a breakthrough treatment designation for the first-line therapy of unresectable HCC which is not amenable to locoregional therapy. A phase III clinical trial of lenvatinib plus pembrolizumab as first-line therapy of unresectable HCC should further confirm the efficacy and safety (NCT03713593) (22). Besides, combination therapy of lenvatinib plus TACE also showed improved ORR (53.3% *vs* 23.3%) (23). The combination therapy of lenvatinib and microwave ablation indicated a better clinical effect with an OS of 14.89 ± 4.89 months and a PFS of 8.65 ± 2.68 months (24).

To conclude, the most prominent feature of lenvatinib would be its noninferiority compared to sorafenib as a first-line drug. The drug’s different, as well as a wider range of targets compared to sorafenib would then mitigate the restrictions of using target therapy given tumor heterogeneity. On-going clinical trials of combination therapies in lenvatinib with antibodies also show better results than that of sorafenib.

#### 2.1.3 Apatinib

Apatinib, or apatinib mesylate (YN968D1), is derived from Valantinib, with a predecessor YN968D11 (N- [4-(1-cyano-cyclopentyl) phenyl]-2-(4-pyridylmethyl) amino-3-pyridine carboxamide mesylate). By occupying the binding site of the VEGFR2, it selectively blocks VEGFR2, thus preventing new blood vessel formation in tumor tissues. In October 2004, Chinese NMPA endorsed apatinib as the third-line treatment for advanced gastric cancer or adenocarcinoma of the gastroesophageal junction. Later in 2020, apatinib by Jiangsu Hengrui is approved for therapies of the second-line treatment in advanced HCC by Chinese NMPA. There exist some adverse events when taking apatinib, but the toxicity is still manageable (25). In terms of combinatorial therapies, the efficacy and safety of apatinib combined with camrelizumab or TACE application showed promising results with improved median PFS, short-term ORR and DCR (NCT03463876, ChiCTR-IPR-17012667, **Table 2**) (26). Therefore, the combination therapy of apatinib and TACE could be a potential treatment for future recurrent HCC patients.

#### 2.1.4 Donafenib

**Figure 3:**
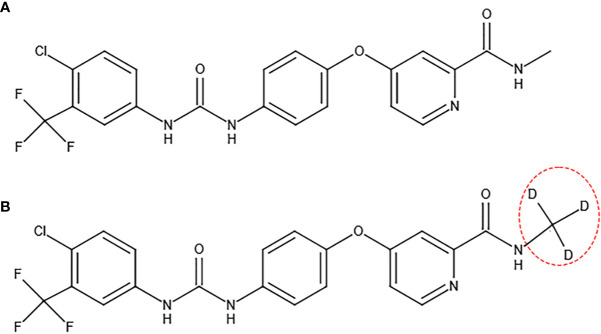
Molecular structures of sorafenib **(A)** and donafenib **(B)**.

Donafenib is formed by replacing the methyl group on a sorafenib molecule with a trideuteriomethyl group (**Figure 3**). By inhibiting phosphorylation of serine/threonine kinases (such as Raf kinase) and by blocking RTK signaling (such as VEGFR and PDGFR), donafenib shows similar antitumor activity as sorafenib for the advanced HCC patients (27). The donafenib group yielded an improved median OS (12.1 months *vs* 10.3 months, p=0.0363) compared to the sorafenib group (**Table 2**) (28). At the same time, the adverse effects of donafenib are fewer than sorafenib (28). According to the Guidelines of Chinese Society of Clinical Oncology (CSCO) on hepatocellular carcinoma that was published in July 2020, donafenib has been listed as a first-line treatment for advanced HCC (29). Donafenib is recommended by the first level specialists with 1A evidence by CSCO (29). On July 9th of 2021, according to Chinese NMPA, donafenib produced by Suzhou Zelgan was approved as a treatment for unresectable HCC patients without systemic therapy.

### 2.2 PDGFR-TKIs

PDGF family consists of PDGF-A to -D polypeptide homodimers and the PDGF-AB heterodimer. They bind to α- and β-tyrosine kinase receptors (PDGFRα and PDGFRβ, respectively) and activates downstream signaling pathways including MAPK, PI3K, phospholipase-γ (PLCγ) and reactive oxygen species (ROS)-dependent STAT3 signaling (30–32). Representative PDGFR-TKIs include regorafenib and sunitinib.

#### 2.2.1 Regorafenib

Despite inhibiting VEGFR1-3, regorafenib inhibits angiogenic kinases PDGFRβ and FGFR1 and the mutant oncogenic kinases c-KIT, RET and B-RAF (33). It is more pharmacologically potent than sorafenib due to a broader spectrum of targets (34). Regorafenib was first approved for metastatic colorectal cancer by the FDA in 2012. In 2017, regorafenib was approved by the FDA as a second-line drug for treating HCC patients previously treated with sorafenib. Regorafenib blocks alpha-fetoprotein (AFP) secretion, further preventing cell migration and invasion in HCC cell lines at different drug doses. A high concentration of regorafenib impedes cell growth in both AFP-positive and AFP-negative HCC cell lines (35, 36). Adverse events of regorafenib are relatively serious yet manageable and clinically acceptable.

#### 2.2.2 Sunitinib

Sunitinib (SU11248) is a low-molecular-weight multi-target TKI that inhibits PDGFRs, VEGFRs, c-KIT, fms-related tyrosine kinase 3 (FLT3), and RET. It was approved to treat imatinib-resistant gastrointestinal stromal tumor (GIST), renal carcinoma, and pancreatic neuroendocrine tumors (37). The most recent clinical trial on sunitinib consists of examining TACE plus sunitinib as first-line therapy in HCC (NCT01164202, **Table 2**) (38). The results showed possible use of TACE plus sunitinib as first-line treatment for patients with HCC who were not candidates for surgical resection.

#### 2.2.3 c-MET TKIs

Hepatocyte growth factor (HGF) binds to c-Met for activation and deregulation, causing its dimerization and autophosphorylation, which further activates the mitogen-activated protein kinase (MAPK), phosphatidylinositol 3-kinase (PI3K), v-src avian sarcoma viral oncogene homolog (Schmidt-Ruppin A-2), and signal transducer and activator of transcription (STAT) signaling pathways (39).

Met constitutive activation may be done either by creating HGF-Met autocrine loops, overexpression of Met, or having activating point mutations in the receptor coding sequence (40). An unusual multi-docking site for MET signals consists of two tyrosine that form a complex with MET when phosphorylated, whose cytoplasmic domain offers additional docking sites for PI3K and SHC. Cell proliferation and transformation are stimulated by increased SHC–GRB2–SOS–RAS pathway activation, whereas cell migration and survival is promoted by selective recruitment of PI3K (41). A representative c-MET-TKIs would be cabozantinib.

#### 2.2.4 Cabozantinib

Cabozantinib, manufactured by Exelixis Inc, was approved by the FDA in 2019 as second-line therapy after treatment of sorafenib or lenvatinib. Cabozantinib is a more potent inhibitor of MET, AXL, RET, FLT3, and TIE-2 than regorafenib. Cabozantinib and regorafenib are structurally similar, yet impose different inhibitory effects on the kinase IC50 (42, 43). Clinical trials of cabozantinib showed promising results especially a recent phase I study (NCT03299946), which showed that cabozantinib plus nivolumab (an immune checkpoint inhibitor) could be an emerging option of neoadjuvant therapy for HCC as reported in 2021 the American Society of Clinical Oncology (ASCO) Symposium (44–46). This was the first clinical trial which investigated a TKI in combination with an immune checkpoint inhibitor in this setting. This pioneer study also provided the first prospective evidence of the above combinations aimed at downstaging HCC. However, this study only included a small patient size (n = 15). Larger sample clinical trials of neoadjuvant approaches are needed in order to confirm the findings in the future.

### 2.3 Other TKIs

The rest of TKIs for HCC targets FGFR (including erdafitinib and orantinib), EGFR (including erlotinib), c-Kit (including anlotinib and pazopanib), JAK (including itacitinib). Among these inhibitors, anlotinib (AL3818) is one of the promising drugs. In May 2018, anlotinib was first approved by Chinese NMPA as a third-line treatment for refractory advanced non-small-cell lung cancer (NSCLC). Then in June 2019, it was approved as a second-line treatment for advanced soft-tissue sarcoma (47). Hypertension, hand-foot skin reaction, fatigue, diarrhea, and anorexia are severe adverse events of anlotinib. In a phase II clinical trial testing the efficacy of anlotinib as first- or second-line treatment for advanced or metastatic HCC patients. Patients without or with prior TKI treatment were divided as cohort 1 or 2. The 12-week PFS rates for cohort 1 and 2 were 80.8% and 72.5%, and the median time to progression were 5.9 months and 4.6 months, respectively. In advanced HCC, anlotinib demonstrated potential effectiveness and safety as a first- or second-line therapy when used in conjunction with a continuous TKIs treatment approach (48). In the treatment for 13 advanced HCC patients with Anlotinib plus AK105, the ORR is 23.3%, and the DCR is 69.2%. At the same time, the adverse events did not exceed level three (49). Therefore, the effectiveness and safety of anlotinib in HCC make it a promising TKIs.

## 3 Comparison of Different TKIs

Because multiple signaling pathways are involved in tumorigenesis and tumor progression, all TKIs for HCC are multi-kinase inhibitors which are designed to target a wide range of targeted kinases. For first-line TKIs, sorafenib targets the serine/threonine kinase Raf or blocks autophosphorylation of multiple RTKs—VEGFR1, 2 and 3, PDGFR, c-Kit, and RET (9). This dual inhibition thus led to satisfactory efficacy with tolerable adverse events. In comparison, lenvatinib is the alternative choice for first-line treatment, but has a different mode of binding compared to sorafenib. When forming complexes with VEGFR2, sorafenib has slow binding kinetics, whereas lenvatinib exhibits a fast association rate constant. It also expresses prolonged residence time than expected due to its delayed dissociation rate constant. Thus, in terms of binding with VEGFR2, lenvatinib is more than 10 times as potent as sorafenib, which offsets sorafenib’s dual inhibition, making lenvatinib confer noninferiority compared to sorafenib (19, 50).

For second-line TKIs, regorafenib, as the standard follow-up after sorafenib treatment, compensates for loss of efficacy by targeting a broader spectrum of targets compared to sorafenib. In addition to common targets such as VEGFR and PDGFR, regorafenib also targets angiogenic receptors TIE2, and oncogenic kinases BRAF V600E, RET, RAF-1, and KIT (34). Moreover, *in vitro* biochemical assays showed that regorafenib is a more potent inhibitor of VEGFR2,PDGFRβ, FGFR1 and c-Kit than sorafenib (43). When compared to regorafenib, cabozantinib offers an alternative choice as they are structurally similar but impose different inhibitory effects on the kinase IC50 (42). While data suggests cabozantinib is a more potent inhibitor of MET, AXL, RET, FLT3, and TIE-2 than regorafenib, it is also more toxic by inducing more frequent adverse events such as palmar-plantar erythrodysesthesia, diarrhea, and asthenia than regorafenib (45). In summary, patients with HCC had limited benefits with single TIK therapies and show better OS with combination therapies. The combination of cabozantinib plus nivolumab was the first step in this direction.

## 4 Nanotechnology as the Potential Delivery System

Emerging nanotechnology as the potential delivery system may improve the therapeutic efficacy of the TKIs, nanoparticle-based TKI delivery techniques are studied nowadays (51). Nanocarriers (NCs) are a form of drug delivery system that is often used to control the pharmacokinetic and pharmacodynamic features of medicines. Nanomaterials used as drug carriers have been shown to improve drug absorption and bioavailability, enhance efficient targeting delivery, prolonged circulation time, and reduce harmful side effects on normal tissues due to their small particle size, large surface area, high surface reactivity and active sites, and desirable adsorption capacity (52).

**Figure 4:**
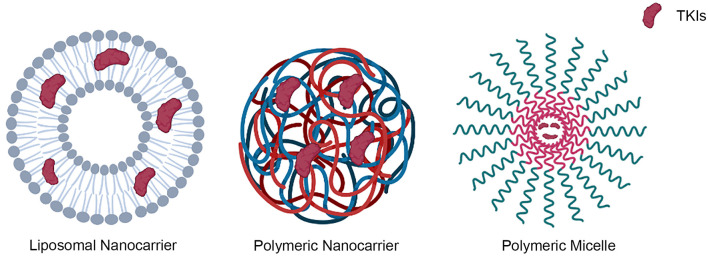
Representative types of nanocarriers that were used in the delivery of TKIs, including liposomal, polymeric and polymeric micelle nano-carriers.

Nanocarriers have been investigated for decades, and the most important carriers in drug delivery were polymeric, liposomal, nonorganic/metal, dendrimer, and micelle nanoparticles. There are multiple novel applications and researches on the delivery of TKIs through nanoparticles on some cancers recently, with most of them being liposomal, polymeric and polymeric micelle nano-carriers (**Figure 4**) (53).

Extracellular vesicles (EVs, 211.4 ± 3.83 nm) could be classified as a type of liposomal nanocarriers. In a research study, they were isolated from human primary adipose-derived stem cells. After incubation and sonication, TKI was loaded into EVs, which was further used to treat radioactive iodine-refractory thyroid cancer cells. The results showed a higher I125 uptake in the TKI treated EVs compared to a TKI-free treatment (54). In another research, polymeric nanoparticles were utilized. BSA-coated, dye-loaded nanoparticles were injected into prostate-specific PTEN/p53-deficient mice pretreated with cabozantinib. Their findings indicate that coating nanoparticles with BSA can improve cabozantinib-induced, neutrophil-mediated targeted intratumoral drug delivery while reducing off-target effects (55). The deliveries of apatinib and cediranib using polymeric nanoparticles were tested for osteosarcoma and glioblastoma, respectively. Both of the studies showed synergistic results compared to individual drugs (56, 57). Moreover, micelle nanoparticles were also studied in a study. A pH-sensitive ester link joins hyaluronic acid (HA) with dasatinib to create the HA-DAS polymer. Then, with rosiglitazone as the core and D-A-tocopheryl polydiethylene glycol isosuccinate (TPGS) and HA-DAS as carriers, a HA-DAS and TPGS mixed micelle system loaded with ROZ was created (THDR-NPs). The developed THDR-NPs showed better efficacy than the taking of free TKIs. In addition, the capacity of THDR-NPs to prevent tumor metastasis has been demonstrated (58). Another study using micelle nanoparticles to deliver anlotinib also showed a more effective uptake to the melanoma cells (59). Polymeric micelles were employed in other three studies relating liver fibrosis, glucose-avid pediatric sarcoma, and melanoma. Involved TKIs include nilotinib, dasatinib and sunitinib, and all of the treatments could enhance the clinical efficacy by utilizing nanoparticles relating to TKIs alone (60–62). Besides previously mentioned methods, electrospray technology was also applied to deliver nintedanib to treat idiopathic pulmonary fibrosis diseases. Higher bioavailability was demonstrated by using this technique (63). The newly developed techniques utilizing nanocarriers showed a higher bioavailability and reduction in the off-target tissues or organs. Noticeably, the bioavailability of nanocarrier involving erlotinib was almost 7 times greater than by orally taking (64).

In the presence of biological barriers *in vivo*, the physicochemical features of these nanocarriers modify their biological identity, which might drastically vary the therapeutic index of their payload and change the desired outcome. Furthermore, the challenges of producing effective medication nanocarriers have resulted in differing perspectives on their safety, permeability of biological barriers, and cellular absorption (65). During *in vivo* and *in vitro* cell exposure, nanoparticles can use a variety of distinct cellular entry pathways to penetrate the plasma membrane, either by endocytosis-based pathways or direct entry to the cells (66). Theoretically nanoparticles could be delivered to our body and focus on the desired target. However, there are only a few clinical studies going on for nanoparticle-based drugs, as the failure rate is high. For further stimulating cell-specific uptake and intracellular endosomal escape of therapeutic molecules, time- and space-controlled release techniques for TKIs delivery systems are necessary, and the responsive or “on-demand” release approach is vital for multidrug administration (51). Therefore, due to the limitations of current orally TKIs drugs, nanoparticle-based TKIs could be a potential way to deal with drug resistance and low bioavailability in future clinical trials, while it requires further investigations and more advanced techniques.

## 5 Molecular Classifications of HCC

Molecular characterization of cancers has contributed to the improved patient outcomes. Recent advances of sequencing technologies have identified the molecular subtypes of HCC (67–74). The correlations between these molecular characterizations of HCC and outcome have been proposed. According to these previous studies, Rebouissou and Nault classified HCC into two major subtypes, “proliferation class” and “non-proliferation class”. The proliferation class is related to HBV, including clinically aggressive tumors that are poorly differentiated. It is distinguished by an enrichment in TP53 inactivating mutations, amplification of FGF19 and CCND1, and frequent activation of pro-survival signaling pathways such as cell cycle, mTOR, RAS-MAPK, and MET. It could be then further divided into “Wnt-TGFβ subclass” and “progenitor subclass,” involving Wnt and TGFβ pathways, and IGF1R and AKT pathways, respectively. Therefore, almost all tyrosine kinases inhibitors except Itacitinib would make contributions to the “proliferation class” of HCC. The non-proliferation class, on the other hand, relates to HCV or alcohol. These HCC cells are more differentiated and have hepatocyte-like characteristics. Non-proliferation class could be divided into “CTNB1 mutation” and “G4,” involving Wnt/β-catenin pathway and IL6/JAK-STAT pathways, respectively (75). Itacitinib would affect this genre as it inhibits the JAK-STAT pathway.

## 6 Biological Alterations in HCC Genesis

Biological alterations in HCC genesis and the impact of TKIs on that pathobiological issues are important for the outcomes (76). Treatment of HCC by TKIs, however, still faces obstacles such as resistance by genetic mutation. For example, changes in the kinase gatekeeper residue may impede inhibitor binding by altering hydrophobic interactions, as suggested by the case of Thr 315 (coded by ACT) mutation in BCR-ABL kinase, which led to imatinib resistance (77). Moreover, overactivation of PI3K would prevent tumor cell from entering kinase-induced apoptosis, which was observed in sorafenib treatment (78). The amplification of the MET gene contributes to PI3K activation as well by driving ERBB3 (HER3)–dependent activation, which causes gefitinib and possible erlotinib resistance (79). Up-regulation of ATP-binding cassette (ABC) proteins can also lead to TKI resistance by exporting inhibitors (80). However, some TKIs such as sorafenib plays a dual role in multidrug resistance as it down-regulates ABCB1 and ABCC2 in HCC, hence opening new therapeutic options for TKI in the treatment of HCC (81). PHGDH was identified as an important driver gene for Sorafenib resistance in HCC by genome-wide CRISPR/Cas9 screening method (82). Although the molecular mechanisms of action of TKIs vary, previous progress have already identified attractive therapeutic approaches for TKI resistant HCC.

## 7 Conclusion

In the past 14 years, treatment of HCC by TKIs has evolved considerably. Although TKIs can delay HCC progression and prolong OS, they still have several challenges: (1) Drug resistance. Random mutations in target receptors lead to abnormal signaling. There are also problems for multi-targeted TKIs where off-target toxicity develops. Heterogeneity in tumor cells could also make TKI less effective due to its specificity of targets (83). The initial sensitivity of patients to TKIs are individually different, as a result, most patients who initially respond well to TKIs will eventually develop drug tolerance. Combination strategies of TKIs plus immune checkpoint inhibitors appearing as a promising strategy to circumvent resistance mechanisms that can be encountered with TKIs, aiming at a synergistic antitumor effect. For future studies, single cell RNA-sequencing could be applied for unraveling the tumor heterogeneity of HCC on a single cell level, further serving as a potential solution to decode the intricate mechanism behind the formation of drug resistance (84). (2) Side effects. Existing TKIs can cause serious side effects while treating HCC (e.g., Sorafenib treatment requires oral high-dose); (3) Low solubility. Only few of the substances could be utilized for targeting by orally taking for TKIs. Nanoparticle-based TKIs delivery techniques would be a promising field for research since they improve the therapeutic efficacy. All the efforts mentioned above will help to overcome TKI resistance and bring better therapy benefit for HCC patients in the future.

## Author Contributions

LM, XT, and BZ drafted the manuscript. YZ, JiaC, YL, YD, ZW, and QL collected data. YS, HZ, JinC, and KT proposed useful comments, suggestions, and revised the manuscript. JD proposed and drew the pictures in this manuscript. LM, YN, and ZP designed the structure and revised the manuscript. All authors contributed to the article and approved the submitted version.

## Funding

This study was supported by Shenzhen Foundation of Science and Technology (grant number JCYJ20170817172116272), Sanming Project of Medicine in Shenzhen (SZSM201812079) and Shenzhen High-level Hospital Construction Fund (2019).

## Conflict of Interest

The authors declare that the research was conducted in the absence of any commercial or financial relationships that could be construed as a potential conflict of interest.

## Publisher’s Note

All claims expressed in this article are solely those of the authors and do not necessarily represent those of their affiliated organizations, or those of the publisher, the editors and the reviewers. Any product that may be evaluated in this article, or claim that may be made by its manufacturer, is not guaranteed or endorsed by the publisher.

## Abbreviations

TKI, tyrosine kinase inhibitor; HCC, hepatocellular carcinoma; OS, overall survival; ORR, overall response rate; PFS, progression-free survival; TACE, transcatheter arterial chemoembolization; RTK, receptor tyrosine kinase.
